# EggLib: processing, analysis and simulation tools for population genetics and genomics

**DOI:** 10.1186/1471-2156-13-27

**Published:** 2012-04-11

**Authors:** Stéphane De Mita, Mathieu Siol

**Affiliations:** 1 Institut de Recherche pour le Développement (IRD), UMR Diversité, Adaptation et Développement des Plantes (DIADE), Montpellier, France; 2 Institut National de la Recherche Agronomique (INRA), UMR Interactions Arbres-Microorganismes (IAM), Nancy, France; 3 Institut National de la Recherche Agronomique (INRA), UMR Amélioration Génétique et Adaptation des Plantes Méditerranéennes et Tropicales (AGAP), Montpellier, France; 4 Institut National de la Recherche Agronomique (INRA), UMR Agroécologie, Dijon, France

## Abstract

**Background:**

With the considerable growth of available nucleotide sequence data over the last decade, integrated and flexible analytical tools have become a necessity. In particular, in the field of population genetics, there is a strong need for automated and reliable procedures to conduct repeatable and rapid polymorphism analyses, coalescent simulations, data manipulation and estimation of demographic parameters under a variety of scenarios.

**Results:**

In this context, we present EggLib (Evolutionary Genetics and Genomics Library), a flexible and powerful C++/Python software package providing efficient and easy to use computational tools for sequence data management and extensive population genetic analyses on nucleotide sequence data. EggLib is a multifaceted project involving several integrated modules: an underlying computationally efficient C++ library (which can be used independently in pure C++ applications); two C++ programs; a Python package providing, among other features, a high level Python interface to the C++ library; and the egglib script which provides direct access to pre-programmed Python applications.

**Conclusions:**

EggLib has been designed aiming to be both efficient and easy to use. A wide array of methods are implemented, including file format conversion, sequence alignment edition, coalescent simulations, neutrality tests and estimation of demographic parameters by Approximate Bayesian Computation (ABC). Classes implementing different demographic scenarios for ABC analyses can easily be developed by the user and included to the package. EggLib source code is distributed freely under the GNU General Public License (GPL) from its website http://egglib.sourceforge.net/ where a full documentation and a manual can also be found and downloaded.

## Background

The exponential growth of sequence databases and the advent of powerful and cost-efficient sequencing technologies have boosted the field of molecular population genetics, providing researchers with an unprecedented and ever growing amount of data [1]. Computing resources appear to be frequently limiting, complicating or even preventing the application of certain analytical methods. To overcome such limitations, automated analysis procedures and efficient computational tools are required.

Although a number of programs and pieces of software implement various tasks routinely performed by population geneticists, few stand-alone packages or libraries gather together a large number into a single framework. Libraries are valuable in several respects. They provide functionalities that can be directly integrated by users in their own programs. It is much easier to modify and extend a library that follows a generic design than a program that was programmed with the aim of fulfilling a single task. Finally, libraries promote code documentation and code re-use. As such, a number of collaborative projects provide the biological science community with open sources projects, such as BioPerl [2], BioJava [3] and Biopython [4]. Among these projects, population genetics are relatively less covered compared with sequence analysis and general purpose computational molecular biology. Thus there is a need for a resource addressing tasks specific to population genetics. As a result of the increase in the amount of available sequence data, even biologists not primarily trained in bioinformatics are faced with tasks requiring programming. Therefore, population genetics/genomics tools should be sufficiently easy to use for non-developers.

In this article we aim at providing the population genetics community with an efficient, flexible, easy to use and complete Python library. The Python programming language combines a clear and intuitive syntax and an extensive standard library, making it suitable for non-experts [5]. We present EggLib, a software package for evolutionary genetics and genomics centered on tools for population genetics analysis. EggLib offers integrated tools for processing biological sequence data, analyzing nucleotide alignments, performing coalescent simulations allowing rarely featured mutation models, mutational bias as well as explicit selfing and estimating demographic parameters through ABC. EggLib aims at complementing the increasingly rich supply of bioinformatics software available to Python users. Besides, we developed the underlying high-performance components as an independent and documented C++ library which can be re-used on its own. In the following of this article, we will briefly describe the architecture of the project by detailing the different components, their content and how they are integrated (Implementation). Then we will provide an overview of the different features of the package and how it compares to existing software in terms of memory usage and running time (Results and Discussion).

## Implementation

**Figure 1:**
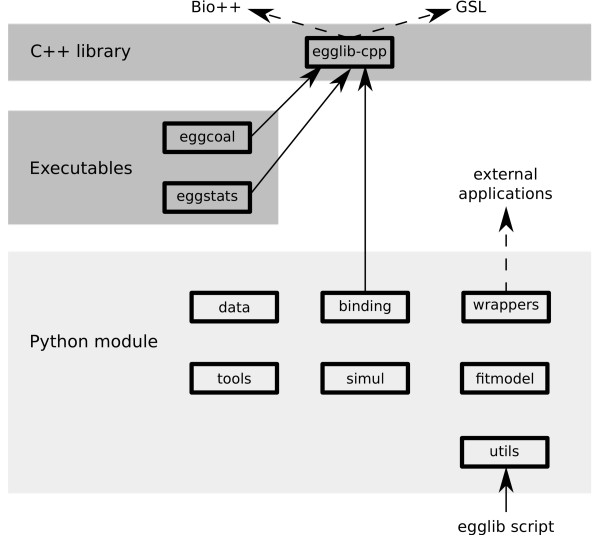
**General architecture and components of the EggLib package**. Solid lines denote dependency relationship (A → B denotes that A depends on, and uses, B). Dashed lines indicate optional dependencies.

EggLib is a composite C++/Python project providing tools for population genetics. The different components are represented on Figure 1. It is based on an underlying C++ library (egglib-cpp) in order to provide efficient tools for sequence storage, analysis, format conversion as well as a coalescent-based simulator. This library can be used in pure C++ applications, and two programs have been derived from it, respectively performing coalescence simulations (eggcoal) and calculating polymorphism statistics on sequence alignments (eggstats). These programs are included in the distributed package. The Python package (egglib-py) fulfills the aim of providing a proficient and intuitive interface of C++ components and extending functionalities with high-level Python classes and functions. Finally, a set of pre-programmed applications relying both on the C++ library and on the Python package are available for interactive execution (thereby behaving as independent programs without having to write any Python code).

The composite nature of EggLib presents several advantages: modularity, simplified maintenance and extendability, and use of the most adapted language for different components. The essential and performance-critical components are implemented in C++. The Python components bring additional features and provide an intuitive and flexible interface. The full content of the different modules is listed in Additional file 1.

### C++ library

egglib-cpp is a fully object-oriented C++ library, meaning that all code is organized in classes, allowing to organize programs in a modular form. egglib-cpp implements tasks related to sequence data storage, simulation and polymorphism analysis. The main classes of the library pertain respectively to aligned and non-aligned set of sequences (Align and Container, respectively), polymorphism analysis (NucleotideDiversity, MicrosatelliteDiversity, HaplotypeDiversity, Fstatistics, HFStatistics) and a coalescent simulator allowing recombination. These classes constitute the backbone of the whole package.

egglib-cpp is available for use in native C++ applications as an independent C++ library package. The functionalities of the C++ library however are available for use in Python applications through the high-level Python interface that is described in the next section.

### Python package

egglib-py is the Python package of EggLib and fulfills several goals, that are reflected by the seven modules it contains (Figure 1). The first module, binding, provides a Python interface of egglib-cpp through a C++-to-Python binding. In most applications, it will not be necessary to handle binding directly, since the other modules are built on top of binding.

The module data contains data storage classes that are likely to be central to most usage of EggLib. The classes dedicated to the management of sequence data (Container and Align) inherit the C++ implementation of their counterparts in egglib-cpp but also incorporates a wide range of interface and extension methods. As a result, the Python versions of Container and Align provide a wide range of functionality transparent with respect to the underlying implementation, such as FASTA import/export, introspection, data access and modification, filtering or extracting. In addition, Align provides several methods for polymorphism analysis. Additional pure Python classes allow to handle microsatellite data (with import/export functions), annotated sequences (incorporating a GenBank Flat File Format parser/formatter) and phylogenetic trees (incorporating a Newick parser/formatter). Similarly to sequence sets, these classes support a wide array of data access, manipulation and edition operations as methods.

The module simul implements coalescent simulations. Since the underlying coalescent simulator is highly flexible, model specifications are passed through two classes (one holding options relative to the demographic model and the other holding options relative to the mutation model) rather than a long, tedious and error-prone argument list. This object-oriented design allows to readily specify complex scenarios.

The module tools includes pure Python components for sequence data manipulation (such as coding sequence translation under various models, open reading frame prediction or alignment concatenation) and extra utilities. The module wrappers provides interfaces to popular applications frequently used by population geneticists such as BLAST + [6], ClustalW [7], MUSCLE [8], PhyML [9], and codeml [10].

The fitmodel module comprises all the classes pertaining to the adjustment of demographic models using Approximate Bayesian Computation (ABC), an increasingly used methodology for demographic inference [11]. Briefly, the principle of ABC is: 1) assume a demographic model that is determined by a set of parameters to be estimated, 2) draw random parameter values from a prior distribution, 3) for each set of parameters, perform a simulation under the assumed demographic model, 4) compare a set of summary statistics computed from the simulated data set to an observed data set, and 5) determine the posterior (estimated) distribution of parameters based on the fit of simulated summary statistics to the observed summary statistics [12,13]. An in-depth description of ABC foundations and methodologies is available in [11]. Compared to existing ABC software, the aim of EggLib is to provide the user with maximal freedom for designing demographic models, statistical priors, and sets of summary statistics. fitmodel has pre-defined models, priors and statistics sets that can be replaced by user-defined classes leveraging all potentialities of EggLib (and beyond). In contrast, low-level analytical steps are implemented in C++ using the GNU Scientific Library in order to maximize performance. Since modern ABC analyses potentially generates very large data sets, files are not fully imported in memory, allowing to accomplish this step using standard workstation computers.

The last module, utils, contains components supporting the interactive commands described hereafter.

### Interactive commands

A program provided in the egglib-py distribution allows to run directly (from a command terminal) a set of pre-programmed commands. These commands are only a subset of what could be achieved with Python programs using EggLib, but they provide a set of immediately available applications. Commands broadly fall into five categories: 1) BLAST-based tools, 2) primer-designing tools, 3) data file conversion or edition, 4) tree manipulation, and 5) ABC estimation of demographic parameters. The latter are the most elaborate. In particular, the command abc_sample performs the steps of coalescent simulation and computation of summary statistics (see the short description of the ABC above), and abc_fit performs the step of estimation of the posterior parameter distribution. In addition, several commands allow to compute marginal or joint posterior distributions, generate graphical plots (using Matplotlib; [14]) and perform posterior simulations using the fitted model as a null model.

### Documentation

The documentation of the C++ classes was generated using Doxygen [15] and that of all Python code was generated using Sphinx [16]. Both Doxygen and Sphinx generate navigable HTML documentation. In addition, a general introduction to EggLib, a manual and description pages have been generated using Sphinx. The whole documentation contains the manual and documentation of both the C++ and Python parts and is available for browsing from http://egglib.sourceforge.net/ and for downloading from the project download page.

## Results and Discussion

In this section, we broadly brush the features offered by EggLib and offer a comparison with widely-used software packages available to the scientific community and offering population genetics utilities. We also compare their performances for file importing and parsing, polymorphism analysis, coalescent simulations and estimation of demographic parameters through ABC. Finally we provide two short examples of code: i) showing a very simple example of polymorphism analysis on a number of loci and ii) explaining how to customize a model for ABC inference using available functions in EggLib.

### Feature overview

**Figure 2:**
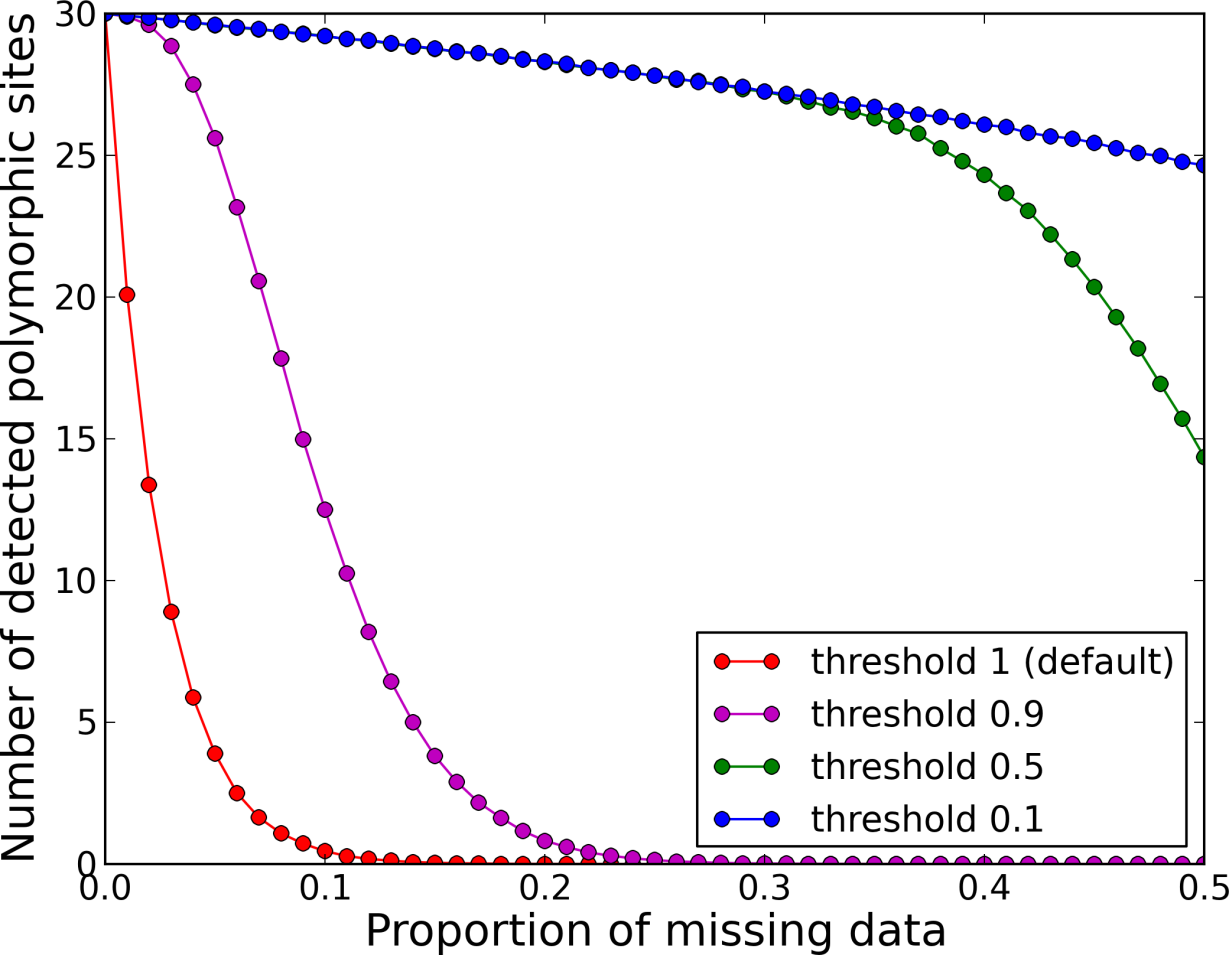
**Effect of missing data and quality threshold on the detection of polymorphic sites**. Estimates of the number of polymorphic sites as a function of the proportion of missing data for different quality thresholds (red = 100%, magenta = 90%, green = 50%, blue = 10%). The simulations parameters are as follow: number of segregating sites = 30; sample size = 40; only polymorphic sites are generated and analyzed; for each value of the proportion of mission data, nucleotides are replaced by N's by random sampling without replacement. Each point represents the average over 5000 repetitions.

An overview of the different type of services provided by Egglib is given in Table 1 and shows whether those features are implemented in other frequently used software. Whereas no general class of features is exclusive to EggLib, the point of EggLib is to bring together most tasks routinely performed in population genetics analyses within a single framework, whenever possible as built-in features (which are efficient and convenient to use). EggLib also brings specific features, such as missing data management and several coalescent simulation options (mutation bias, explicit position of markers, diploid model with selfing). Missing data (and alignment gaps) are a recurrent concern of empirical studies. EggLib can perform nucleotide diversity analyses allowing a given proportion of missing data (the statistics are computed on the remaining data). The power of this approach to detect polymorphic sites that would be otherwise ignored is depicted in Figure 2.

**Table 1:** Features available in EggLib and alternative population genetics software packages

	EggLib	Biopython	PyCogent	Bio++	DnaSP	ms	CoaSim	DiyABC	ABCToolbox	msABC	ABCreg
**Reference**	This paper	[4]	[17]	[18]	[19]	[20]	[21]	[22]	[23]	[24]	[25]
**Sequence data management**											
Input format	FASTA + converters	Many formats	Many formats	Many formats	Several formats			Genepop format	Specific format		Tabular data
Alignment	Available (wrappers)	Available (wrappers)	Available (wrappers)								
Storage model	Full storage in memory	Full storage (alignments) and iterative parsing	Full storage in memory	Full storage in memory	Full storage in memory						

**Sequence analysis**											
BLAST wrapper	Available	Available	Available								
Gene prediction			Available								

**Diversity analysis**											
Microsatellites	Built-in	Genepop wrapper									
Sequences	Built-in			Built-in	Built-in	From simulations					
Coding sequences	With Bio++			Built-in							

**Phylogenetics**	Distance and maximum-likelihood methods through wrappers		Built-in distance and maximum likelihood methods + wrappers	Built-in distance and maximum likelihood methods							

**Simulations**											
Coalescence (standard model)	Built-in and ms wrapper	ms wrapper			Available	Available	Available	-			
Recombination	Available	Available			Available	Available	Available				
Structured models	Available	Available			Available	Available					
Diploid samples & selfing	Available										
Infinite-site model	Available	Available				Available	Fixed number of sites				
Homoplasy	Available	Available					Available				
Microsatellite models	Available	Available					Available				
Output	Sequences, FASTA, trees, statistics, Python objects	Arlequin-compatible file			P-values	Sequences, statistics	Sequences, Python objects				

**ABC inference**											
Models	Pre-defined models + all models allowed by the simulator (not restrictive)							Customizable divergence models with population size changes	Depends on the simulator used	All models allowed by ms	
Summary statistics	Pre-defined statistics sets + all statistics available in EggLib (not restrictive)							Microsatellite and within- and between-population sequence statistics	Calculated by simulator or provided by the user	Within- and between-population sequence statistics	
Analysis method	Rejection and local-linear regression							Rejection and local-linear regression	Rejection, local-linear regression, generalized linear models and others		Rejection and local-linear regression

### Usage of egglib-py

**Figure 3:**
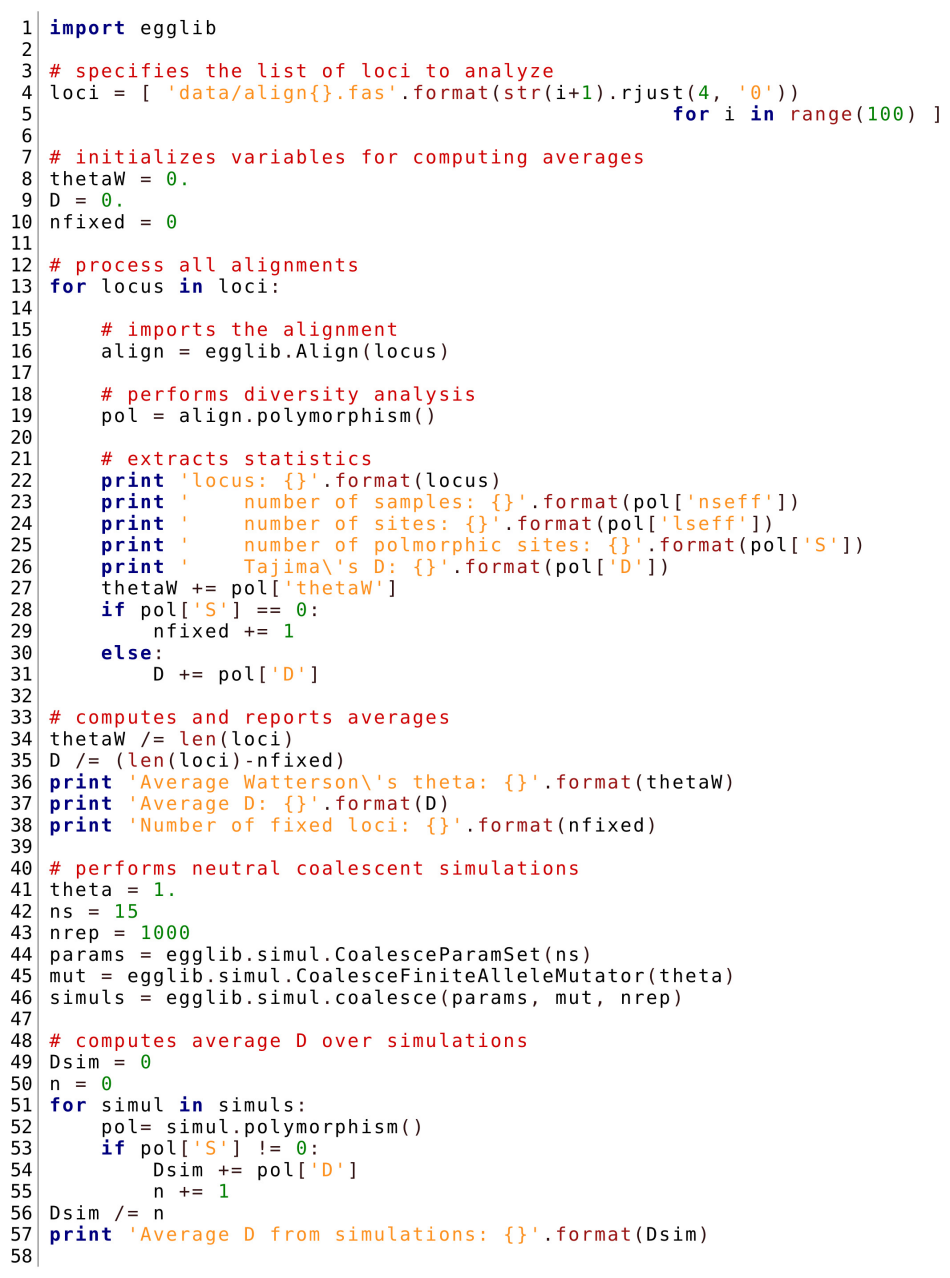
**Example of diversity analysis implemented in Python using egglib-py**. This script imports 100 FASTA-formatted alignments, performs a basic diversity analysis and finally compares the average Tajima's *D *statistic to a number of neutral coalescent simulations under the standard model. Lines 16, 19, and 44-46 are commented in the text. All operations are performed using the Align class and the simul module of egglib-py (full documentation is included in the reference manual available online).

The programming interface of the Python package EggLib was designed to be intuitive, simple to use, and to allow fast development of scripts automating population genetics analyses. This was done by providing high-level interface layers above components implemented in C++ and internalizing much of the complexity. We present a simple example to demonstrate how a data set comprising an arbitrary number of loci can be analyzed in a compact and readable fashion by combining Python and EggLib simple syntax (Figure 3). The example's comments describe what each block achieves (a full documentation of EggLib's class Align and simul module is available in the online reference manual). Here, we will point out the parts of the code exploiting EggLib's potentialities. Line 16 (align = egglib.Align(locus)) creates an alignment instance. The user is only required to specify the name of the FASTA file containing the alignment (locus). Line 19 (pol = align.polymorphism()) performs a polymorphism analysis with default settings, that correspond to the standard approach. One of the options (not shown here, see the reference manual) allows to support missing data (see above and Figure 2). The returned value, pol, is a dictionary (associative array), that allows straightforward access to computed statistics. Whenever several populations and/or outgroup sequences are present in the alignment, between-population and outgroup-based statistics will be automatically computed. Finally, lines 44-46 demonstrate the usage of the coalescent simulator. In this example, the simplest possible model is used: a single constant-sized population with an infinite-site model of mutation. Three steps are performed: creation of a CoalesceParamSet instance (specifying the number of samples; line 44), creation of a FiniteAlleleMutator instance (specifying the mutator type and the rate of mutation; line 45), and, finally, call to the coalesce function that returns a list of Align instances (line 46). The advantage of this three-step syntax for configuring coalescent simulation is that it can accommodate both simple models (as the one used here) and more complex scenarios exploiting all potentialities of the coalescent simulator.

### User-defined ABC model

Several commands accessible from the command line utilities of EggLib allow one to perform ABC analysis using command-line tools. However, the set of pre-defined models cannot be exhaustive and one of our aims is to allow using all EggLib functionalities to design any possible demographic model.

**Figure 4:**
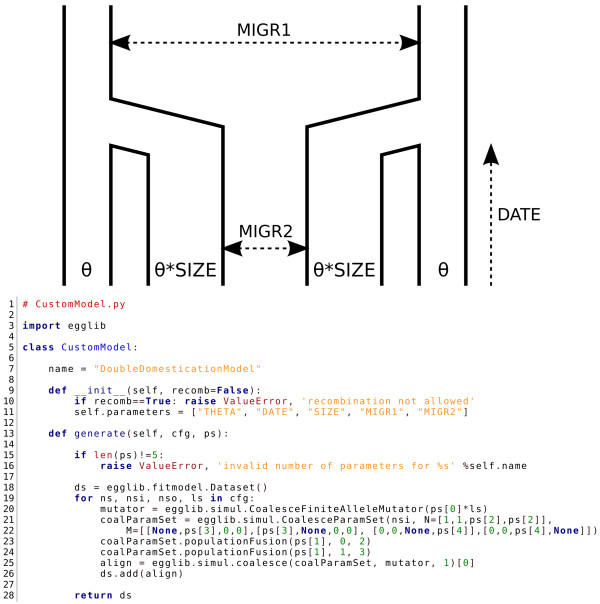
**Code example: User-defined ABC model**. Example of user-defined demographic model extending EggLib's pre-implemented ABC models. A graphical representation of the model is showed at the top of the picture, and the code to implement it is showed at the bottom. Explanations can be found in the main text.

The model presented in Figure 4 is an arbitrary example of model that is not available in the fitmodel module. This model is depicted at the top of the figure. It has five different parameters: *THETA *(θ in the picture), *DATE, SIZE, MIGR1 *and *MIGR2*. This model can be viewed as a double, simultaneous domestication from two partially isolated stocks (time runs from top to bottom). *DATE *is the age of the domestication event, the migration parameters specify exchange rates *MIGR1 *and *MIGR2 *between pairs of populations and *SIZE *gives the relative size of cultivated populations.

The code at the bottom of Figure 4 shows the implementation of this model within the EggLib framework. Note that ABC models should conform to a few requirements: they must be formalized as a class; they must define their name and the names of all parameters; their constructor must accept at least one argument specifying whether recombination must be implemented (and deal with it appropriately), but it can accept more arguments; and they must contain a generate method which specifies the body of the model implementation. This very piece of code can be used in conjunction to fitmodel (within a Python script), but it can also be used to add this model to the list of models available through the interactive command abc_sample. Custom priors and sets of summary statistics can be incorporated using a similar system, although currently (as of version 2.1.2) abc_sample does not currently support run-time addition of priors and summary statistics (such support is planned).

The generate method is the hook that connects the model to the rest of the ABC framework. It must take two arguments: a sample configuration and a set of parameter values drawn from the prior (the fitmodel documentation provides details of the exact format of these data). The generate method must return simulated data sets (using a type defined in fitmodel). Apart from these constraints, the user has full freedom with regard to what is actually done for generating the data set. Obviously, all potentialities of the coalescent simulator incorporated within EggLib are allowed. Furthermore, all forms of post-processing operations are not only possible, but easy to implement using egglib-py. For example, one can readily include error rates or sampling or ascertainment biases and set them as model parameters.

### Performance

The running time and maximum memory usage of programs performing common population genetics operations using EggLib compared with alternatives (whenever available) is shown on Tables 2, 3, 4 and 5. All tests were run on a laptop computer and (except for coalescent simulations) were repeated 10 times. Tests were performed using EggLib 2.1.2, Biopython 1.58, libsequence 1.7.4 [26], analysis 0.8.1 (containing compute, polydNdS and rsq) [26], the version of ms updated December 11, 2009 [20], coasim-python 1.3 [21], msABC 20111219 [24] and ABCreg 2009-07-30 [25] (which were all the latest available versions at time of testing). For coalescent simulations (including in ABC), EggLib was set to use at most 4 processor cores.

**Table 2:** Running time and memory use while importing FASTA files

File	EggLib		Biopython	
	
	Time (s)	Memory (MB)	Time (s)	Memory (MB)
Large alignment (96.5 MB)	2.19	115.5	2.48	129.6

*Oryza sativa *coding sequences (92.5 MB)	2.39	100.4	5.12	313.8

*Oryza sativa *pseudomolecules (361.0 MB)	7.83	396.4	11.49	401.0

Note: The large alignment contains 10,000 sequences of 10,000 bp. The coding sequences of the *Oryza sativa *genome represent 67,393 sequences ranging from 153 to 16,311 bp while its pseudomolecules represent 12 sequences ranging from 23,011,239 to 43,268,879 bp.

**Table 3:** Running time and memory use while performing diversity analyses

File	EggLib		libsequence	
	Time (s)	Memory (MB)	Time (s)	Memory (MB)
1000 files (49.8 MB) minimal	4.17	9.3	-	-
1000 files (49.8 MB) standard	9.54	9.5	12.34	1.8
1000 files (49.8 MB) LD	26.43	151.7	47.87	124.8
1 file (33.0 MB) minimal	4.35	104.0	-	-
1 file (33.0 MB) standard	6.84	92.6	2.63	44.1
1 file (6.0 KB) coding	0.16	8.7	0.06	0.1

Note: We analyzed 1000 simulated alignments of 50 sequences (plus one outgroup) of 1000 bp and a single alignment of 7 sequences of 4,920,321 bp. A subset of this alignment containing 6 sequences of 999 bp was analyzed for coding statistics. The minimal set of statistics was the number of polymorphic sites, θ estimators and Tajima's *D*. The standard set of statistics included minimal statistics plus haplotype-based statistics. Linkage disequilibrium (LD) was computed between polymorphic sites. For coding sequences, non-synonymous and synonymous θ estimators were calculated (for EggLib, the functions of Bio++ are called).

**Table 4:** Running time and memory use while performing coalescent simulations

Model	Egglib		ms		CoaSim	
	Time (s)	Memory (MB)	Time (s)	Memory (MB)	Time (s)	Memory (MB)
standard	7.68	48	1.27	43	16.67	80
recombination	8.77	53	1.99	44	16.45	79
structured	7.65	48	1.50	42	20.75	79

Note: All three models (standard, recombination and structured) have 40 sequences with a fixed number of mutations of 100. 10,000 repetitions were run for each model. For the model with recombination, the scaled recombination parameter was set to 5 for all programs and the number of recombining segments was set to 1000 for eggcoal and ms (CoaSim does not require this parameter). For the structured model, 4 populations of 10 samples with a migration rate of 1 were simulated. The populations joined 10 coalescent time units in the past.

**Table 5:** Running time and memory use while performing ABC

Simulation step	Egglib		msABC	
Model + summary statistics	Time (s)	Memory (MB)	Time (s)	Memory (MB)
SNM + SDZ	13.71	25.6	7.24	8.9
SNMR + SDZ	27.09	55.6	26.10	8.8
PEMR + SDZ	16.72	44.8	13.46	8.6
BNM + SDZ	15.68	37.6	8.27	9.1
IM + SDZ	40.06	70.3	21.52	14.2
AM + SDZ	25.11	57.8	*	*
SNM + SFS	15.83	25.8	-	-
SNMR + SFS	29.85	55.6	-	-
PEMR + SFS	18.05	44.9	-	-
BNM + SFS	18.22	36.5	-	-
IM + SFS	46.94	63.6	-	-
AM + SFS	29.15	51.6	-	-

**Analysis step**			**ABCreg**	

Data file: 830 MB	70.82	131.0	30.74	628.7

Note: Models: standard neutral model (SNM), standard neutral model with recombination (SNMR), population expansion model with recombination (PEMR), bottleneck model (BNM), island model with two populations (IM), admixture model (AM). Uniform prior bounds: 0-0.05 (per site) for the mutation and recombination rates, 0.01-1 for the migration rate, 0-1 for date/duration parameters, 0-1 for the population size during bottleneck 0-10 for the ancestral population size. Summary statistics sets: SDZ (number of polymorphic sites, Tajima's *D *and Fay and Wu's *H*), SFS (site frequency spectrum with 8 categories). The SFS was available only with EggLib. 20 loci of 40 sequences 1000 bp-long were analyzed and each ABC simulation run generated 1000 data samples. For the analysis phase, a large data set of 5,000,000 samples (containing two varying model parameters and nine statistics) was used. EggLib was used through the interactive commands abc_sample and abc_fit. (*) The AM model could not be implemented with msABC.

EggLib is comparatively more efficient than Biopython for importing large FASTA files (Table 2). The Align class of EggLib is slightly more efficient than AlignIO of BioPython for importing a large alignment. For importing data files representing the whole *Oryza sativa *genome, the Container class of EggLib is much more efficient than SeqIO of Biopython (EggLib is able to import these two files fully in memory in a few seconds and with a limited memory overhead: the memory use is hardly larger than the file size). However, the difference between EggLib and Biopython reflects a difference in paradigm (import all file at once for EggLib, and read sequence one at a time for Biopython).

The comparison of an EggLib script for analyzing polymorphism with programs developed using the C++ libsequence library (compute for standard statistics, polydNdS for coding sequence statistics and rsq for linkage disequilibrium) shows that skipping unneeded statistics can significantly fasten the analysis. For analyzing a single alignment, libsequence programs are better, but for processing many alignments in a row a single loop using EggLib is more efficient. In EggLib, the linkage disequilibrium analysis is comparatively more efficient, and the coding sequence analysis (based on the wrapping of Bio++) is comparatively less efficient.

The comparison of the eggcoal, ms and CoaSim simulators shows that ms is consistently and significantly the fastest and the least memory-demanding (Table 4). eggcoal lies between ms and CoaSim. EggLib has a generic design that makes it difficult to maximize performance, explaining part of the discrepancy. However, we believe that future versions will improve performance, especially thanks to improved implementation of recombination and multithreading scheme that are currently planned.

We compared the performances of EggLib commands for ABC to the very efficient programs msABC (for the simulation phase) and ABCreg (for the analysis phase). We used two different summary statistics sets: SDZ (number of polymorphic sites, Tajima's *D *and Fay and Wu's *H*) and SFS (site frequency spectrum with 8 categories). The SFS was available only with EggLib. EggLib was used through the interactive commands abc_sample and abc_fit. We found that EggLib abc_sample was slower than msABC and used more memory (chiefly because of Python-level multithreading). This is explained by the performance of the original ms program (see above), that was efficiently leveraged in msABC. However, the overhead tends to be decreased compared with the eggcoal/ms comparison presented before, showing that the EggLib integration does not worsen performance. We therefore expect that future improvements of the coalescent simulator will bring EggLib closer to the level of msABC. For the analysis step, a large data file of 5,000,000 samples was imported and analyzed. We observed that this step of the ABC procedure was not limiting in running time (compared to the simulation step) but could be limiting in memory use. Therefore we followed a strategy favoring data access from file, which is relatively slower but more memory efficient. EggLib and ABCreg are therefore complementary regarding the speed/memory balance.

### Prospects

EggLib is under active development and we expect new features to be added in the future. Our current routes for improving the package include: improving the performance of the coalescent simulator thanks to a new design of the recombination process and an improved parallelization scheme; easing the definition by users of custom ABC models, sets of summary statistics and priors using automated helpers; improving the performance of the ABC framework by internalizing replications within the C++ layer and removing unnecessary steps (such as Align conversion) without interfering with the general flexibility of the framework; putting a special effort in documentation, especially by providing tutorials besides the complete reference manual.

## Conclusion

EggLib has been actively developed for several years, both at C++ and Python levels. It has been thoroughly tested, with a special emphasis for the accuracy of the computation of diversity statistics, coalescent simulations and ABC, both against theoretical expectations and/or available software, whichever available. It was successfully compiled and installed on GNU/Linux, MacOS X, Windows NT under both Cygwin and MinGW/MSYS. EggLib has been available for public download and used since July 2008 (initially under the name SeqLib) and the total number of downloads was over 1,500 by December 2011. EggLib has been used in published research [27-30] and has also been integrated in the SNP analysis pipeline SNiPlay as a module for computing diversity statistics [31]. This illustrates that EggLib might be used by developers as well as non-developers. The design of the package allows software developers to use underlying tools as population genetics routines. Other projects (such as SNiPlay) can fulfill the task of providing graphical user interface software to end users, but its simple Python syntax and the utils command-line tools make possible to use EggLib and leverage its functionalities without expert programming skills.

## Availability and requirements

**• Project name: **EggLib

**• Project home page: **http://egglib.sourceforge.net/

**• Operating system: **platform-independent

**• Programming languages: **C++ and Python

**• Other requirements: **Python 2.x (2.6 or higher); optional dependencies on external software for some functionality

**• License: **GNU General Public License version 3 (+ CeCILL Free Sofware License for pre-compiled packages)

**• Any restrictions to use by non-academics: **none

## Competing interests

The authors declare that they have no competing interests.

## Authors' contributions

SDM and MS planned the project, wrote and tested the code, maintain the project, wrote the manuscript and approved its final version. All authors read and approved the final manuscript.

## Supplementary Material

Additional file 1 **Content of EggLib C++ library and Python package**. List of all classes and functions defined in EggLib, and brief description. Function names are followed by brackets. In EggLib, class names are capitalized and function names are not. The class methods are not indicated in this table. For those, consult the online documentation. Click here for file

Additional file 2 **Available polymorphism statistics**. List of statistics returned by diversity analysis methods of the Align and SSR classes. When results are reported as a dictionary, the list of available keys is reported. The file contains, whenever appropriate, a description of the conditions under which the statistics are computed, and bibliographic references. Click here for file
