# Supplementary material for: EggLib: processing, analysis and simulation tools for population genetics and genomics
Source: BMC Genet. 2012 Apr 11;13:27. doi: 10.1186/1471-2156-13-27 (PMC3350404; doi:10.1186/1471-2156-13-27)
Supplement: Additional file 1 — Content of EggLib C++ library and Python package. List of all classes and functions defined in EggLib, and brief description. Function names are followed by brackets. In EggLib, class names are capitalized and function names are not. The class methods are not indicated in this table. For those, consult the online documentation. [file 1471-2156-13-27-S1.PDF]

EggLib: processing, analysis and simulation tools for population genetics and genomics

Additional file 1 – Content of EggLib C++ library and Python package

egglib-cpp

Exception types

EggArgumentValueError	Exception type for argument value errors
EggException	Base exception type for errors occurring in *egglib-cpp*
EggFormatError	Exception type for file/string formatting errors
EggInvalidCharacterError	Exception type for invalid character (in diversity analysis)
EggMemoryError	Exception type for memory errors
EggOpenFileError	Exception type for errors while opening a file
EggRuntimeError	Exception type for runtime errors
EggUnalignedError	Exception type for unaligned sequences

Data storage or manipulation

Align	Handles a sequence alignment
CharMatrix	Interface for classes usable as a square matrix of characters
Consensus	Generates consensus sequences
Container	Handles a set of sequence alignment (aligned or not)
Convert	Performs conversion between sequence holder types
DataMatrix	Matrix of data as integer values
Fasta	FASTA parser/formatted
Ms	ms-like sequence format parser
Staden	Parser of Staden output format

Diversity analysis

BaseDiversity	Base class of diversity classes
BppDiversity	Computes diversity statistics using third-party library Bio++
FStatistics	Computes F_{IS}, F_{ST} and F_{IT} from diploid data
HaplotypeDiversity	Computes diversity based on haplotype analysis
HFStatistics	Computes F_{ST} and F_{IT} from haploid data
LinkageDisequilibrium	Analyzes linkage disequilibrium per pair of polymorphic sites
MicrosatelliteDiversity	Analyzes microsatellite data
NucleotideDiversity	Analyzes of sequence data alignment
SitePolymorphism	Implements diversity analysis at the site level

Coalescent simulator

Arg	Ancestral recombination graph
Bottleneck	Bottleneck event

Change	Pure virtual base class for parameter changes
Controller	Controls a coalescent simulation
Current	Represents the current set of populations
Edge	Edge of the ancestral recombination graph
Mutation	Very simple container of some information relative to a mutation
Mutator	Implements mutation models
ParamSet	Set of parameters
Population	Handles a single population

Coalescent demographic event types

AllMigrationRateChange	Change of the migration rate of all population pairs
AllPopulationSizeChange	Change of the size of all populations
GrowthRateChange	Change of the growth rate of all populations
PopulationBottleneck	Population-specific bottleneck event
PopulationFusion	Fusion of two populations
PopulationGrowthRateChange	Change of a single population's growth rate
PopulationParamChange	Single parameter changes applied to a single population
PopulationSplit	Split of a population
SelfingRateChange	Change of the selfing rate
SingleMigrationRateChange	Change of a single migration rate
SingleParamChange	Pure virtual base class for single parameter changes
SinglePopulationSizeChange	Change of a single population size

Other

ABC	Model estimation by Approximate Bayesian Computation
Random	Pseudo-random number generator

egglib-py

data module

Container	Unaligned sequence set
Align	Aligned sequence set
SequenceItem	Type used while iterating over sequence sets
SSR	Simple sequence repeats set
TIGR	TIGR annotation format
GenBank	GenBank annotated sequence format
GenBankFeature	GenBank annotation feature
GenBankFeatureLocation	GenBank annotation feature location
Tree	Phylogenetic tree
TreeNode	Phylogenetic tree node

tools module

aln2fas()	Format conversion
staden()	Format conversion
get_fgenesh()	Format conversion

genalys2fasta()	Format conversion
Mase	Mase alignment format
LD()	Compute linkage disequilibrium
backalign()	Nucleotide alignment based on protein alignment
concat()	Concatenates alignments
longest_orf()	Finds longest open reading frame
rc()	Reverse complements sequences
translate()	Protein translation
ungap()	Removes positions with gaps in alignment
GeneticCodes	Database for genetic codes
compare()	Ambiguity codes aware sequence comparison
motifs()	Finds repeated motifs in a sequence
locate()	Finds a motif in a sequence
chisquare()	Performs a chi-square test
correl()	Computes correlation coefficients
ranges()	Identifies continuous ranges
ReadingFrame	Handles reading frame positions.
Updater	Monitor progress of long-running tasks
wrap()	String formatting tool

wrappers module

ms()	Interface to ms coalescent simulator
BLAST	Interface to BLAST program
BL2SEQ	Interface to BLAST program for pairwise comparison
BLASTdb	Handles a BLAST database
clustal()	Interface to ClustalW multiple alignment program
muscle()	Interface to Muscle multiple alignment program
phyml()	Interface to PhyML maximum likelihood phylogeny software
nj()	Interface to Phylip neighbor-joining phylogeny software
Codeml	Interface to codeml program of the PAML package
Primer3	Interface to Primer3_core primer detection program

coalesce module

coalesce()	Python interface to the underlying C++ coalescent simulator
CoalesceParamSet	Handles coalescent parameters
CoalesceMutator	Base class for mutator types
CoalesceFiniteAlleleMutator	Represents a mutation model with fixed number of alleles
CoalesceInfiniteAlleleMutator	Represents a mutation model with an infinite number of alleles
CoalesceStepwiseMutator	Represents the stepwise mutation model
CoalesceTwoPhaseMutator	Represents the two-phase mutation model

fitmodel module

Dataset	Manages a set of read or simulated alignments
ParamSample	Holds a list of parameters
import_posterior()	Imports a posterior file
PriorParseError	Handles exception raised by incorrect prior distribution
PriorDiscrete	Implements discrete prior distributions

PriorDumb	Implements continuous prior distributions
SNM	Standard neutral model
PEM	Population expansion model
BNM	Bottleneck model
GDB	Composite-parameter bottleneck model
GGDB	Generalized composite-parameter bottleneck model
IM	Island model
IMn	IM with different population sizes
IMG	IM with exponential growth
IMiG	IMG with independent rates in each population
IMiGn	IMiG with different population sizes
SM	Split model
AM	Admixture model
MRC	Migration rate change model
DOM	Domestication model
TPH	Statistics set: thetaW, Pi, He (averaged over all loci)
TPS	Statistics set: total thetaW, Pi for each population, and Hudson's Snn
SFS	Statistics set: site frequency spectrum
JFS	Statistics set: joint frequency spectrum

utils module

abc_bin	Binarizes a posterior distribution
abc_compare	Compares different models
abc_fit	Fits model using either rejection-sampling or local linear regression
abc_plot1D	Plots marginal posterior distributions
abc_plot2D	Plots a bivariate posterior distributions
abc_psimuls	Performs posterior simulations
abc_sample	Generates samples for ABC
abc_statsdisc	Properties of a discretized posterior distribution
abc_statsmarg	Marginal properties of a posterior distribution
analyzer	Extended port of samplestat
blastgb	Blasts all coding sequences from a GenBank file
clean_seq	Removes ambiguity characters from nucleotide sequences
clean_tree	Removes internal labels and branch lengths in a newick tree
codalign	Protein-based alignment of coding sequences
concat	Concatenation of sequence alignments
concatgb	Concatenation of GenBank records
consensus	Builds consensus of sequences with matching names
cprimers	Finds consensus primers
extract	Extract specified ranges of an alignment
extract_clade	Extracts the sequences corresponding to a tree clade
family	Finds homologs of a gene family using BLAST
fasta2mase	Converts a fasta alignment to the mase format
fasta2nexus	Converts a fasta alignment to the NEXUS format
fasta2phyml	Converts a fasta alignment to the \`phyml\` format
fg2gb	Generates a GenBank record from fgenesh output
gb2fas	Converts GenBank records to fasta

infos	Displays basic information from fasta files
interLD	Computes linkage disequilibrium statistics between two loci
matcher	Finds homologous regions between two sequences
names	Lists sequence names from a fasta file
phyml	Performs maximum-likelihood phylogenetic reconstruction
rename	Rename sequences according to a replacement list
reroot	Changes the orientation of a newick tree
select	Selects a given list of sequences from a fasta file
sprimers	Design copy-specific PCR primers from an alignment
staden2fasta	Converts a STADEN GAP4 dump file to fasta
translate	Translates coding sequences to protein sequences
truncate	Truncates sequence names
ungap	Removes gaps from a sequence alignment
winphyml	Computes tree likelihood along a sliding window
