# Supplementary material for: EggLib: processing, analysis and simulation tools for population genetics and genomics
Source: BMC Genet. 2012 Apr 11;13:27. doi: 10.1186/1471-2156-13-27 (PMC3350404; doi:10.1186/1471-2156-13-27)
Supplement: Additional file 2 — Available polymorphism statistics. List of statistics returned by diversity analysis methods of the Align and SSR classes. When results are reported as a dictionary, the list of available keys is reported. The file contains, whenever appropriate, a description of the conditions under which the statistics are computed, and bibliographic references. [file 1471-2156-13-27-S2.PDF]

EggLib: processing, analysis and simulation tools for population genetics and genomics

Additional file 2 – Available polymorphism statistics

This document lists all items returned by polymorphism functions of *egglib-py*. Unless otherwise stated, polymorphism functions return a dictionary. The content of returned dictionaries is dynamic. Statistics that are not always available are marked by one of the following codes:

[A] Available if lseff>0;
[B] Available if S>0;
[C] Available if skipAllHaplotypeStats=False;
[D] Available if skipHaplotypeDifferentiationStats=False;
[E] Available if npop>1;
[F] Available if skipDifferentiationStats=False;
[G] Available if npop=3;
[H] Available if skipOutgroupBasedStats=False;
[I] Available if an outgroup is available;
[J] Available if So>0;
[K] Available if Tv>0;
[L] Available if eta>0;
[M] Available for coding sequences.

Bibliographic references are cited using numbers and are given at the end of this document.

Note: θ is the population parameter $4N\mu$ where $2N$ is the population size and μ the mutation rate, except in the return value of *SSR.Fstats()* where it is the between-population component of genetic variance.

Align.polymorphism()

nseff	Average number of effectively analyzed sequences
lseff	Number of effectively analyzed sites
npop	Number of populations
S	Number of polymorphic sites
eta	Minimum number of mutations
singletons	List of position of singleton sites
sites	List of polymorphic sites as SitePolymorphism instances
siteIndices	List of position of polymorphic sites
thetaW	Watterson's estimator of θ [1][A]
Pi	Nucleotide diversity [A]
D	Tajima's *D* [2][B]
He	Haplotypic diversity [C]
K	Number of haplotypes [C]

alleles	Haplotype index for all sequences [C]
Fst	Population differentiation based on nucleotides [3][B][C][D][E]
Gst	Population differentiation based on haplotypes [4][B][C][D][E]
Hst	Population differentiation based on haplotypes [4][B][C][D][E]
Kst	Population differentiation based on nucleotides [4][B][C][D][E]
Snn	Nearest neighbor statistic [5][C][D][E]
average_Pi	Average of π among populations [A][E][F]
pop_Pi	List of π for all populations [A][E][F]
pair_CommonAlleles	Number of alleles in common per pair of populations [E][F]
pair_FixedDifferences	Number of fixed differences per pair of populations [E][F]
pair_SharedAlleles	Number of shared polymorphisms per pair of populations [E][F]
pop_Polymorphisms	Number of polymorphic sites per population [E][F]
pop_SpecificAlleles	Number of specific alleles per population [E][F]
pop_SpecificDerivedAlleles	Number of specific alleles (only derived) per population [E][F]
CommonAlleles	Total number of alleles in common in one pair of populations [E][F]
FixedDifferences	Total number of fixed differences between two populations [E][F]
SharedAlleles	Total number of shared polymorphisms between two populations [E][F]
SpecificAlleles	Total number of population-specific alleles [E][F]
SpecificDerivedAlleles	Total number of population-specific alleles (only derived) [E][F]
triConfigurations	Frequency of the 13 possible site configurations for 3 populations [F][G]
lseffo	Number of analyzed oriented sites [H][I]
So	Number of oriented polymorphic sites [H][I]
thetaH	Fay and Wu's estimator of θ [6][H][I][J]
thetaL	Zeng *et al.*'s estimator of θ [7][H][I][J]
H	Fay and Wu's *H* [6][H][I][J]
E	Zeng *et al.*'s *E* [7][H][I][J]
Z	Standardized version of *H* [7][H][I][J]

Align.BPPpolymorphism()

S	Number of polymorphic sites
Sinf	Number of parsimony-informative sites
Ssin	Number of singleton sites
eta	Minimum number of mutations
thetaW	Watterson's estimator of θ [1]
He	Nucleotide diversity
T83	Tajima's estimator of θ [8]
Ti	Number of transitions
Tv	Number of transversions
TiTv	Transition/transversion ratio [K]
K	Number of distinct haplotypes
H	Haplotypic diversity
D	Tajima's *D* [2][B]
Deta	Tajima's *D* based on eta [2][L]
Dflstar	Fu and Li's *D** (without outgroup) [9][L]
Fstar	Fu and Li's *F** (without outgroup) [9][L]
rhoH	Hudson's estimator of ρ [10]
Dfl	Fu and Li's *D* [9][L][I]
F	Fu and Li's *F* [9][L][I]
Sext	Number of mutations on external branches [I]
ncodon1mut	Number of codon sites with exactly one mutation [M]
NSsites	Number of non-synonymous sites [11][M]
Ssites	Number of synonymous sites [11][M]
nstop	Number of codon sites with a stop codon [M]
nsyn	Number of codon sites with a synonymous change [M]
SNS	Number of non-synonymous polymorphic sites [M]
SS	Number of synonymous polymorphic sites [M]
tWNS	Watterson's estimator of θ computed on non-synonymous sites [1][M]
tWS	Watterson's estimator of θ computed on synonymous sites [1][M]
PiNS	Nucleotide diversity computed on non-synonymous sites [M]
PiS	Nucleotide diversity computed on synonymous sites [M]
MK	McDonald-Kreitman test table [12][I][M]
NI	Neutrality index [13][I][M]

Align.Rmin() (returns an integer)

-	Minimum number of recombination events [14]

Align.matrixLD()

n	Number of pairs of sequences
d	Nested dictionary of the distance between pairs of polymorphic sites
D	Nested dictionary of the *D* statistic for pairs of polymorphic sites [15]
Dp	Nested dictionary of the *D*' statistic for pairs of polymorphic sites [16]
r	Nested dictionary of the *r* statistic for pairs of polymorphic sites [17]
r2	Nested dictionary of the r^2 statistic for pairs of polymorphic sites [17]

SSR.stats()

k	Number of alleles
V	Variance of allele size
He	Expected heterozygosity
thetaI	Estimator of θ under the infinite allele model [18]
thetaHe	Estimator of θ under the stepwise mutation model, based on H_E [19]
thetaV	Estimator of θ under the stepwise mutation model based on *V* [20]

SSR.Fstats() (returns a tuple of three values)

-	Weir and Cockerham's estimator of *f* (F_{IS}) [21]
-	Weir and Cockerham's estimator of θ (F_{ST}) [21]
-	Weir and Cockerham's estimator of *F* (F_{IT}) [21]

Bibliographic references

1 Watterson G: **On the number of segregating sites in genetical models without recombination.** *Theoretical Population Biology* 1975, **7**:256-276.

2 Tajima F: **Statistical method for testing the neutral mutation hypothesis by DNA polymorphism.** *Genetics* 1989, **123**:585-595.

3 Hudson RR, Slatkin M, Maddison WP: **Estimation of levels of gene flow from DNA sequence data.** *Genetics* 1992 **132**:583-589.

4 Hudson RR, Boos DD, Kaplan NL: **A statistical test for detectig geographical subdivision**. *Molecular Biology and Evolution* 1992, **9**:138-151.

5 Hudson RR: **A new statistic for detecting genetic differentiation**. *Genetics* 2000, **155**:2011-2014.

6 Fay JC, Wu C: **Hitchhiking under positive Darwinian selection**. *Genetics* 2000, **155**:1405-1413.

7 Zeng K, Fu YX, Shi S, Wu CI: **Statistical tests for detecting positive selection by utilizing high-frequency variants**. *Genetics* 2006, **174**:1431-1439.

8 Tajima F: **Statistical method for testing the neutral mutation hypothesis by DNA polymorphism**. *Genetics* 1989, **123**:585-595.

9 Fu Y, W-H Li: **Statistical tests of neutrality of mutations**. *Genetics* 1993, **133**:693-709.

10 Hudson RR: **Estimating the recombination parameter of a finite population model without selection**. *Genetical Research* 1987, **50**:245-250.

11 Nei M, Gojobori T: **Simple methods for estimating the numbers of synonymous and nonsynonymous nucleotide substitutions**. *Molecular Biology and Evolution* 1986, **3**:418-426.

12 McDonald JH, Kreitman M: **Adaptive protein evolution at the *Adh* locus in *Drosophila***. *Nature* 1991, **351**:652-654.

13 Rand DM, Kann LM: **Excess amino acid polymorphism in mitochondrial DNA: Contrasts among genes from *Drosophila***. *Molecular Biology and Evolution* 1996; **13**:735-748

14 Hudson RR, N L Kaplan: **Statistical properties of the number of recombination events in the history of a sample of DNA sequences**. *Genetics* 1985, **111**:147-164.

15 Lewontin RC, Kojima K: **The evolutionary dynamics of complex polymorphisms**. *Evolution* 1960, **14**: 458-472.

16 Lewontin RC: **The interaction of selection and linkage. I. General considerations; heterotic models**. *Genetics* 1964, **49**:49-67.

17 Hill, WG, Robertson A: **Linkage disequilibrium in finite populations**. *Theoretical and Applied Genetics* 1968, **38**: 226- 231.

18 Ewens WJ: **The sampling theory of selectively neutral alleles**. *Theoretical Population Biology* 1972, **3**:87-112.

19 Ohta T, Kimura M: **A model of mutation appropriate to estimate the number of electrophoretically detectable alleles in a genetic population**. *Genetical Research* 1973, **22**:201-204.

20 Moran PA: **Wandering distributions and the elecrophoretic profile**. *Theoretical Population Biology* 1975, **8**:318-330.

21 Weir BS, Cockerham CC: **Estimating *F*-statistics for the analysis of population structure**. *Evolution* 1984, **38**:1358-1370.
